# Design and Fabrication of Ultrathin Metallic Phase Shifters for Visible and Near-Infrared Wavelengths

**DOI:** 10.3390/mi16010074

**Published:** 2025-01-10

**Authors:** Qing Guo, Jinkui Chu, Chuanlong Guan, Chuxiao Zhang, Ran Zhang

**Affiliations:** 1 State Key Laboratory of High-Performance Precision Manufacturing, Dalian University of Technology, Dalian 116024, China; gqdlut@mail.dlut.edu.cn (Q.G.); guan357@mail.dlut.edu.cn (C.G.); zchuxiao@mail.dlut.edu.cn (C.Z.); zhangr@dlut.edu.cn (R.Z.); 2 Key Laboratory for Micro/Nano Technology and System of Liaoning Province, Dalian University of Technology, Dalian 116024, China; 3 Ningbo Institute of Dalian University of Technology, Ningbo 315016, China

**Keywords:** nanoimprint lithography, nanowire gratings, oxygen plasma ashing process, transmittance

## Abstract

The polarization state of light is critical for biological imaging, acousto-optics, bio-navigation, and many other optical applications. Phase shifters are extensively researched for their applications in optics. The size of optical elements with phase delay that are made from natural birefringent materials is limited; however, fabricating waveplates from dielectric metamaterials is very complex and expensive. Here, we present an ultrathin (14 nm) metallic phase shifter developed using nanoimprinting technology and the oxygen plasma ashing technique for visible and near-infrared wavelengths. The fabrication process can produce desirable metallic phase shifters with high efficiency, large area, and low cost. We demonstrate through a numerical simulation and experiment that the metallic phase shifter exhibits phase delay performance. Our results highlight the simplicity of the fabrication process for a metallic phase shifter with phase delay performance and offer important opportunities for creating high-efficiency, ultrathin polarizing elements, which can be used in miniaturized devices, such as integrated circuits.

## 1. Introduction

Polarization is one of the important properties of optical radiation, and polarized states are crucial for realizing various optical applications, particularly in industry and scientific research. Polarized states are decisive in complex application scenarios such as biological imaging [1,2], acousto-optics [3], bio-navigation [4], and microscopy techniques [5,6]. Optical elements that provide phase delay are key components of many optical systems. They are usually made of natural birefringent materials, which limit their miniaturization and integration. Quarter-waveplates and linear-to-circular polarization conversion are linear retarders with a 90° phase retardance between two orthogonal linear eigenpolarizations. Quarter-waveplates and linear-to-circular polarization conversion are commonly used for circularly polarized light production [7,8,9,10,11,12,13,14,15,16,17,18,19]. Additionally, ultrathin subwavelength polarization transformers have garnered considerable interest from numerous researchers [20,21,22]. Recently, metamaterials with subwavelength spacing and dimensions have displayed unusual electromagnetic properties, providing a powerful boost for implementing different electromagnetic wave operations, such as perfect absorption, flat lensing, holograms, and polarization transformation [23,24,25,26]. Metamaterials with subwavelength two-dimensional arrays could enhance electromagnetic fields and control light polarization states, making them attractive for optical system integration and miniaturization [27,28,29]. However, the structures of dielectric metamaterials generally have a large aspect ratio [30,31,32,33,34]. While combining metal nanowire strips and dielectrics could effectively improve the birefringence of metamaterials, the fabrication process is very complicated and expensive.

In this study, a metamaterial with phase delay functionality is presented and fabricated, which is capable of achieving high transmittance and an approximately 90° phase delay in the visible and near-infrared wavelengths (VIS-NIR). Additionally, the fabrication process of an ultrathin metallic phase shifter based on nanoimprint lithography (NIL) and the oxygen plasma ashing process is proposed [35]. This process eliminates the electron beam lithography and metal etching processes in favor of applying NIL. Consequently, the fabrication process is simplified with the benefits of low cost, high efficiency, and large area. Theoretical analysis reveals that the structure has a large fabrication tolerance error; therefore, mass manufacturing is possible.

## 2. Design and Simulation

**Figure 1:**
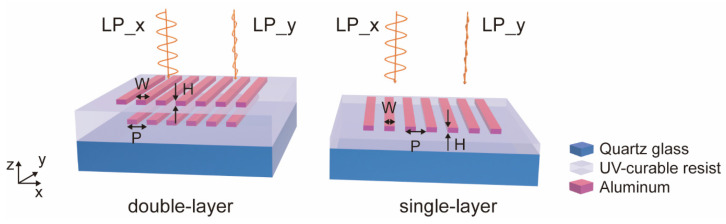
Structural schematics of double-layer and single-layer aluminum nanowire gratings.

We chose aluminum for the fabrication of an ultrathin metallic phase shifter at the subwavelength scale, particularly because of its good linear polarization properties and fabrication simplicity. In this article, the finite difference time domain (FDTD) method was applied to simulate the performance of aluminum nanowire gratings in VIS-NIR. We fabricated the ultrathin metallic phase shifter from single-layer aluminum nanowire gratings. We also conducted a simulation analysis of the transmittance and phase for both double-layer and single-layer aluminum nanowire gratings. The structural diagrams of the double-layer and single-layer aluminum nanowire gratings are shown in Figure 1, where the period P is 180 nm. In this model, aluminum nanowire gratings were placed on a quartz glass substrate with an embedded UV-curable resist. Additionally, we applied periodic boundary conditions to the unit cell.

**Figure 2:**
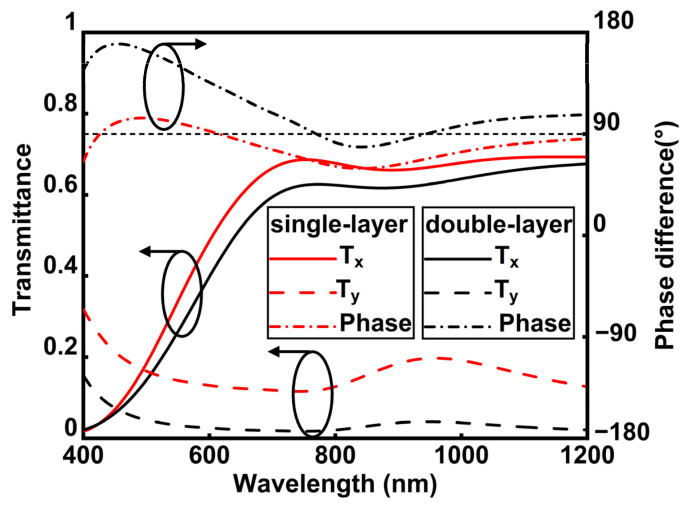
Transmittance and phase of double-layer and single-layer aluminum nanowire gratings.

As illustrated in Figure 2, we calculated the transmission spectra of single-layer and double-layer aluminum nanowire gratings for linearly polarized light. The aluminum nanogrids with anisotropic structures produce plasmonic resonances at different wavelengths depending on the polarization state of incident light [36]. Furthermore, a weak transmission dip occurs around 900 nm due to the intrinsic optical loss of aluminum. It is verified that aluminum nanowire grids lead to non-resonant and broadband anisotropic transmission in VIS-NIR. For x-axis-polarized light (LP_x), which is perpendicular to the nanowire gratings, the transmission rate of the single-layer aluminum nanowire gratings is slightly higher than that of the double-layer aluminum nanowire gratings. Similarly, for y-axis-polarized light (LP_y), which is parallel to the nanowire gratings, the single-layer aluminum nanowire gratings also demonstrate a slightly higher transmission rate than the double-layer aluminum nanowire gratings. Furthermore, the double-layer aluminum nanowire gratings exhibit poorer performance in achieving a 90° phase difference effect compared to the single-layer aluminum nanowire gratings. To achieve a phase delay of 90°, we opted to fabricate the single-layer aluminum nanowire grid to accomplish this phase difference.

**Figure 3:**
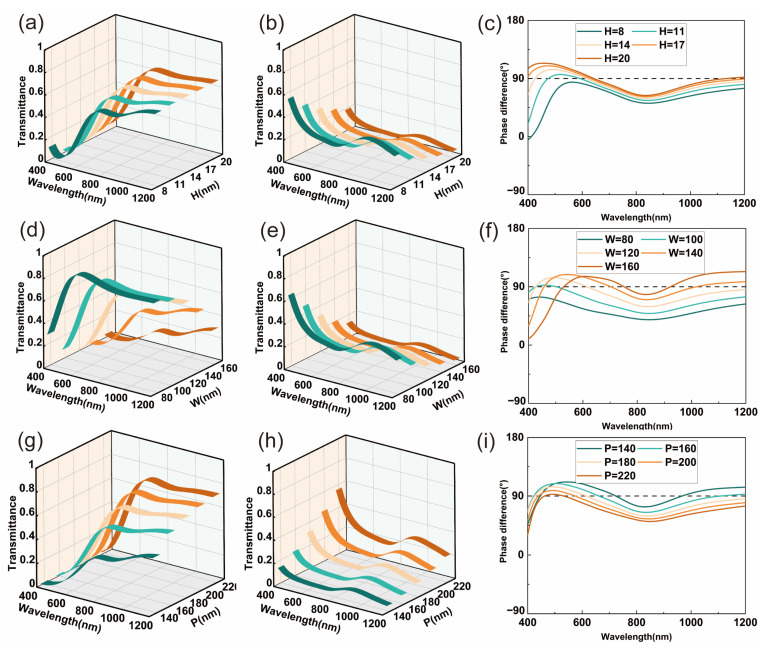
Effect of thickness, width, and period on transmittance and phase. (**a**) Effect of thickness on transmittance of *x*-polarized light. (**b**) Effect of thickness on transmittance of *y*-polarized light. (**c**) Effect of thickness on phase. (**d**) Effect of width on transmittance of *x*-polarized light. (**e**) Effect of width on transmittance of *y*-polarized light. (**f**) Effect of width on phase. (**g**) Effect of period on transmittance of *x*-polarized light. (**h**) Effect of period on transmittance of *y*-polarized light. (**i**) Effect of period on phase.

To enhance nanogrid performance, we scanned the design parameters of the single-layer aluminum nanowire gratings, including grid thickness, grid width, and period. The fabrication process is much simpler and has a higher success rate since it does not require aluminum metal etching and photolithographic alignment, which are very challenging for the nanofabrication of feature size down to tens of nanometers. Figure 3a,b illustrate the transmittance of *x*- and *y*-polarized light corresponding to the variation in thicknesses, where P = 180 nm, and W = 120 nm. As displayed in Figure 3a,b, it can be observed that the *x*-polarized light transmittance becomes larger, while the *y*-polarized light transmittance decreases with increasing thickness. However, we can observe that the phase difference at thickness H = 14 nm is closer to 90° in Figure 3c. Conversely, it can be observed that a slight change in thickness does not induce an excessive change in the phase difference. Figure 3d,e display the transmittance of *x*- and *y*-polarized light corresponding to the variation in widths, where P = 180 nm, and H = 14 nm. As depicted in Figure 3d,e, it can be observed that the *x*-axis-polarized light transmittance decreases, while that of the *y*-axis decreases with increasing width. However, we can observe in Figure 3f that the phase difference at width W = 120 nm is closer to 90°. Figure 3g,h illustrate the transmittance of *x*- and *y*-polarized light corresponding to the variation in periods, where H = 14 nm, and W = 120 nm. As displayed in Figure 3g,h, it can be observed that the *x*-polarized light transmittance increases, while that of *y*-polarized light decreases with an increase in period. However, we can observe that the phase difference at P = 180 nm is closer to 90° from Figure 3i.

According to Figure 3, we scanned the thickness, width, and period at subwavelength scales as H = 14 nm, W = 120 nm, and P = 180 nm, respectively. This configuration enables the single-layer aluminum nanowire gratings to exhibit a standard phase difference of 90° at wavelengths of λ = 427 nm and λ = 615 nm. The single-layer aluminum nanowire gratings boast an exceptionally small thickness and provide a phase delay of approximately 90°. The ultrathin single-layer aluminum nanowire gratings demonstrate phase delay performance that is primarily grounded in the theory of self-complementary metamaterials [37]. However, the assumption that enables a wide bandwidth in self-complementary metamaterials relies on the existence of an infinitely thin perfect conductor. Consequently, the ultrathin single-layer aluminum nanowire gratings have not fully achieved the ideal 90° phase delay performance across a wide bandwidth. These gratings are capable of converting linearly polarized light into elliptically polarized light, making them suitable for studies related to elliptical polarization.

**Figure 4:**
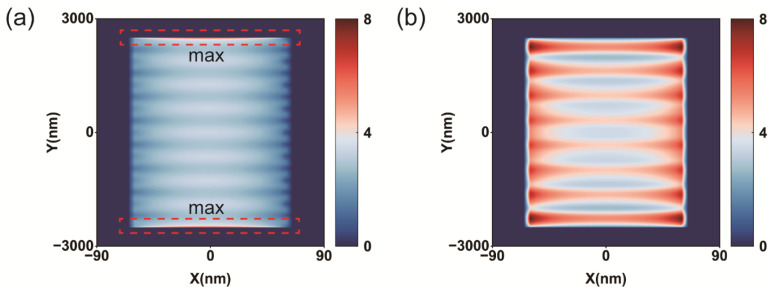
Near-field distribution. (**a**) Incident polarization is along the length of the nanowire gratings. (**b**) Incident polarization is along the width of the nanowire gratings. The wavelength used in the simulation is 427 nm.

To understand the underlying mechanism of the aluminum nanowire grating structure, we calculated the normalized near-field distribution of polarized light; the simulation results are illustrated in Figure 4. We observed unique near-field distribution for different incident polarized light. For linearly polarized light incidents along the *y*-axis, the electric field intensity was mostly concentrated at the edges of the aluminum nanowire gratings (Figure 4a). Conversely, for linearly polarized light incidents along the *x*-axis, the electric field intensity was mostly localized in the aluminum nanowire gratings (Figure 4b).

For incident light with an electric field vector oriented in parallel with the aluminum nanowire gratings (i.e., the *y*-axis in Figure 1), it is almost completely reflected by the metal wire gratings. For incident light with an electric field vector oriented vertically to the aluminum nanowire gratings (*x*-axis in Figure 1), it is mostly transmitted by the metal wire gratings. For linearly polarized light incident along the *y*-axis, the aluminum nanowire gratings allow current to flow in a nearly infinite length on the *y*-axis. For linearly polarized light incident along the *x*-axis, it easily passes through the aluminum nanowire gratings without strong resonance in the wavelength range. Therefore, the aluminum nanowire gratings exhibit non-resonant properties over a wide range of wavelengths of *y*-polarized light, but they do not interact much with *x*-polarized light.

## 3. Device Fabrication and Characterization

**Figure 5:**
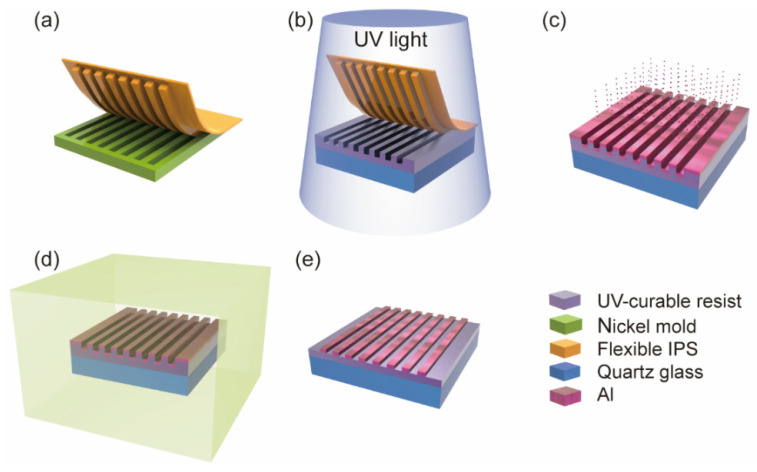
Schematic diagram of fabrication process: (**a**) preparation of flexible IPS by NIL; (**b**) transfer patterns of IPS to UV-curable resist layer using UV-NIL; (**c**) aluminum thermal evaporation process; (**d**) oxygen plasma ashing process; (**e**) desirable ultrathin metallic phase shifter.

**Figure 6:**
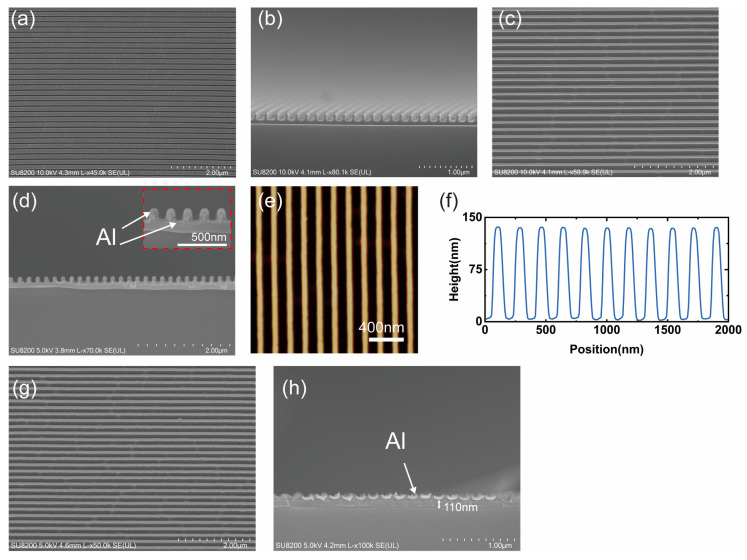
Manufacturing process of ultrathin metallic phase shifter: (**a**) SEM surface view of flexible IPS; (**b**) SEM cross-sectional image and (**c**) SEM surface view of feature patterns of UV-curable resist layer; (**d**) SEM cross-sectional image of double-layer aluminum nanowire gratings; (**e**) AFM surface view and (**f**) AFM cross-sectional image of double-layer aluminum nanowire gratings; (**g**) SEM cross-sectional image and (**h**) SEM surface view of ultrathin metallic phase shifter.

The fabrication process of an ultrathin metallic phase shifter with 90° phase delay performance is illustrated in Figure 5. A 4-inch nickel mold was fabricated in advance using electron beam lithography. The entire surface of the mold was patterned with nanoscale wire gratings, characterized by P = 180 nm, W = 60 nm, and H = 80 nm. Using an Eitre 6 nanoimprinter (Obducat AB, Lund, Sweden), a thermal nanoimprint process was employed to fabricate the intermediate polymer stamp (IPS). The feature patterns on the IPS were completely complementary to those on the nickel mold (see Figure 5a). The scanning electron microscope (SEM) surface view of the flexible IPS is displayed in Figure 6a, which illustrates that the feature patterns on the nickel mold were completely replicated.

For easy characterization, we used a 2-inch silicon substrate, which was rotationally coated at 2000 rotations per minute to form the 210 nm UV-curable resist layer and then baked at 90 °C for 120 s. The transfer of the feature patterns from the flexible IPS to the UV-curable resist layer was accomplished by UV-NIL on the Eitre 6 nanoimprinter. The air pressure was set at 40 bar, while the imprinting temperature was 160 °C for 120 s. The demolding process was completed at 115 °C, while the UV exposure process was conducted for the light curing of the UV-curable resist layer (see Figure 5b). A SEM cross-sectional image and SEM surface view of the feature patterns of the UV-curable resist layer are illustrated in Figure 6b,c, respectively, where the transferred patterns can be observed clearly from the cross-sectional views.

Subsequently, the aluminum thermal evaporation process was performed on the samples after placing them in a Model 400 vacuum coater (COSTAR, Nanyang, China) to deposit an approximately 14 nm aluminum layer. Due to the concave and convex structure of nanowire grids, the deposited aluminum forms double-layer aluminum nanowire gratings (see Figure 5c). A SEM cross-sectional image of the double-layer aluminum nanowire gratings is displayed in Figure 6d, which reveals that the aluminum nanowire gratings fabricated by the aluminum thermal evaporation process have superior consistency. As found using atomic force microscopy (Figure 6e,f), it is also observed that the consistency of the bilayer aluminum nanowire grids is excellent.

Finally, we placed the double-layer aluminum nanowire gratings in a PLUTO-F plasma surface treatment system (PLUTOVAC, Shanghai, China) to obtain an ultrathin metallic phase shifter using the oxygen plasma ashing process. The plasma surface treatment system operated at 300 W for 180 s. Based on the isotropic characteristics of the oxygen plasma ashing process, we used the protruding part of the grid of the UV-curable resist layer as a sacrificial material and removed the overlaying aluminum, consequently completing fabrication (Figure 5d,e). As found using SEM (Figure 6g,h), an ultrathin metallic phase shifter was successfully fabricated, and the images displayed good consistency. SEM cross-sectional images illustrated that the profiles are not perfectly rectangular due to the deformation of the flexible IPS during the imprinting process and the instability of the deposition process in the aluminum thermal evaporation process. Additionally, the fabrication process eliminated the metal etching and alignment processes, which greatly simplified the process and improved manufacturing efficiency.

**Figure 7:**
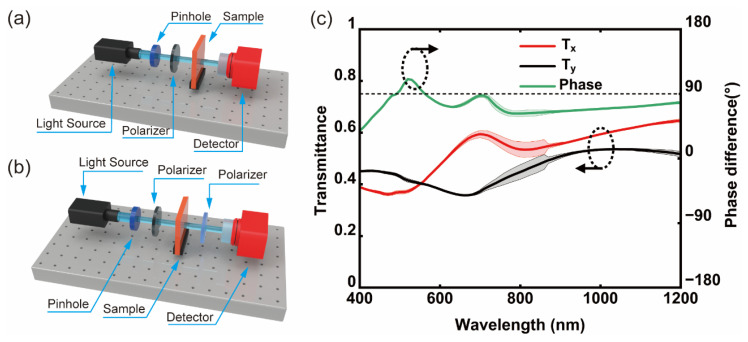
Test results of transmittance and phase of ultrathin metallic phase shifter: test system schemes of (**a**) transmittance and (**b**) phase; (**c**) test results of transmittance and phase difference.

We fabricated an ultrathin metallic phase shifter on a quartz glass substrate using the fabrication process depicted in Figure 5. To characterize performance, we tested its transmittance and phase using a Lambda 1050+ UV/VIS/NIR spectrophotometer (PerkinElmer, Waltham, MA, USA), and systematic diagrams are displayed in Figure 7a,b [38,39]. The test results reveal that the transmittance of *x*-polarized light is mainly distributed between 0.36 and 0.65, while the transmittance of *y*-polarized light is mainly distributed between 0.35 and 0.54, and the phase difference is mainly distributed between 38° and 111°. Especially at λ = 490 and 580 nm, the desirable performance of 90° phase delay was observed. The test results exhibit significant fluctuations around 860 nm, primarily due to the change in the detectors of the spectrophotometer. The difference between the test data and the simulation calculation is mainly because of manufacturing errors such as the thermal deformation of the flexible IPS, the directional control of aluminum thermal evaporation, and the demolding process of NIL.

## 4. Conclusions

In this work, we simulated the transmittance of and phase difference in both double-layer and single-layer aluminum nanowire gratings based on the FDTD method in VIS-NIR and obtained anharmonic and broadband anisotropic transmittance. Simulations revealed that the ultrathin metallic phase shifter satisfies the characteristics of 90° phase delay. By scanning dimensions at the subwavelength scale, we obtained the target parameters of H = 14 nm, W = 120 nm, and P = 180 nm while obtaining the desirable 90° phase difference performance at λ = 427 and 615 nm. The ultrathin metallic phase shifter was well suited for practical applications regarding cost and mass production, especially because of the practical accessibility of aluminum and the simplicity of the fabrication process. Additionally, the near-field distribution was calculated to understand the underlying mechanisms of the nanowire grid. The simple, highly efficient, and large-area fabrication of a metallic phase shifter with 90° phase delay properties was presented using NIL and the oxygen plasma ashing process. Furthermore, the fabricated aluminum nanogrids exhibited superior consistency. The fabrication process was simplified by eliminating the metal etching and alignment processes, and fabrication efficiency was substantially improved. Our experimental and theoretical analyses revealed that a metallic phase shifter can be obtained by the fabrication process conducted in this paper. However, fabrication quality can be improved by investigating flexible IPS deformation, the aluminum thermal evaporation process, and the demolding process of NIL.

## Data Availability

Data are contained within this article.
